# Primary Cardiac Angiosarcoma with Right Atrial Rupture

**DOI:** 10.1093/icvts/ivaf227

**Published:** 2025-10-01

**Authors:** Yusuke Nakata, Kazuyuki Miyamoto, Kenichi Nishiyama, Seiya Kato

**Affiliations:** Department of Cardiovascular Surgery, Fukuoka Red Cross Hospital, Fukuoka City, Fukuoka 815-8555, Japan; Department of Cardiovascular Surgery, Fukuoka Red Cross Hospital, Fukuoka City, Fukuoka 815-8555, Japan; Department of Pathology, Fukuoka Red Cross Hospital, Fukuoka City, Fukuoka 815-8555, Japan; Department of Pathology, Saiseikai Fukuoka General Hospital, Fukuoka City, Fukuoka 815-8555, Japan

**Keywords:** angiosarcoma, cardiac tamponade, coronary arteries, fistula, right atrium, rupture

## Abstract

A 47-year-old man presented with a recurrent refractory bloody pericardial effusion. Based on the preoperative imaging and pericardiocentesis results with negative cytology, we concluded that blood from a coronary fistula caused the repeated cardiac tamponade. However, pathological findings of the right atrial tissue confirmed primary cardiac angiosarcoma. In this report, we present a unique case as it involves a preoperatively or intraoperatively undetected tumour and a primary cardiac angiosarcoma that induced a fatal outcome, emphasizing the importance of early therapeutic intervention.

## INTRODUCTION

Primary cardiac angiosarcoma (PCA) is a rare malignancy with nonspecific features mimicking other cardiac tumours, making early diagnosis challenging, and distant metastases are frequently present at diagnosis. We report a case of PCA complicated by preoperatively or intraoperatively unidentified right atrial rupture.

## CASE REPORT

**Figure 1.**
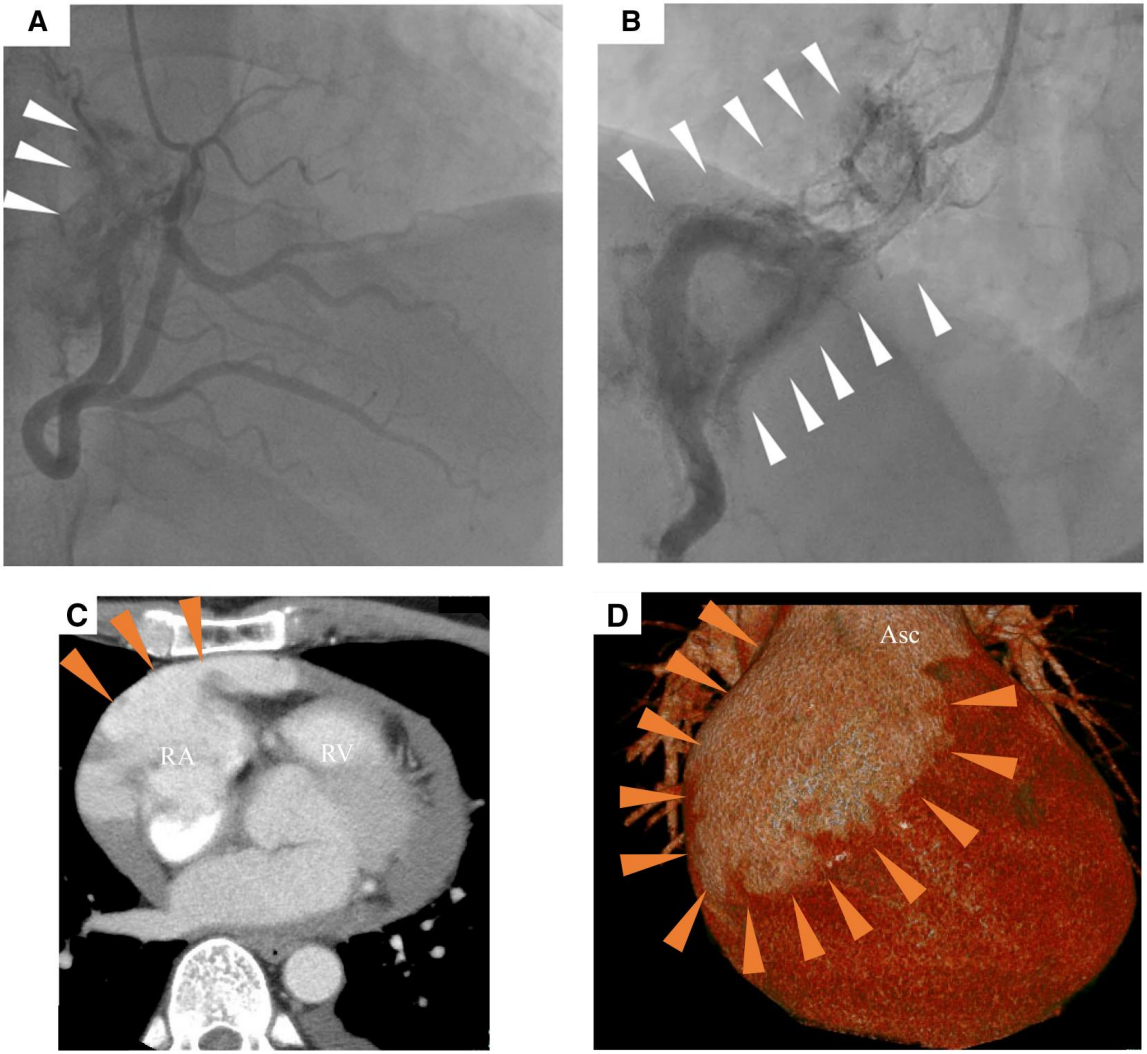
(A) Coronary angiography (CAG: left anterior oblique) presenting collateral vessels from the right coronary artery (RCA) to the right atrium (RA) (white arrows). (B) During the third cardiac tamponade, the CAG displays further RA enlargement with structural collapse, without RCA stenosis (white arrows). (C) Contrast-enhanced computed tomography reveals an enlarged RA and RA rupture-induced massive pericardial effusion due to RA rupture (red arrows). (D) Three-dimensional computed tomography reveals an RA rupture-induced hematoma and RA tissue, extending to the front of the ascending aorta (red arrows) RA, right atrium; RV, right ventricle; Asc Ao, Ascending Aorta

A 47-year-old male presented with chest discomfort, bilateral leg oedema, and weight gain over 3 months. Echocardiography revealed a large pericardial effusion, which was drained (590 mL). While no early re-accumulation of pericardial fluid was subsequently observed, pericardial effusion recurred at 1 month and again at 2 months after the initial drainage. Cytology and cultures were repeatedly negative. During the first episode, echocardiography and computed tomography revealed a haematoma surrounding the right atrium (RA). In subsequent episodes, coronary angiography revealed collateral vessels from the right coronary artery (RCA) to the RA (**Figure 1A and B**). During the third episode, computed tomography indicated RA enlargement and hematomas (**Figure 1C and D**) with new nodular lung shadows that had not been present the preceding month. Blood from a ruptured coronary artery fistula between the RCA and RA was identified as the cause of the tamponade, leading to surgery without further examination.

**Figure 2.**
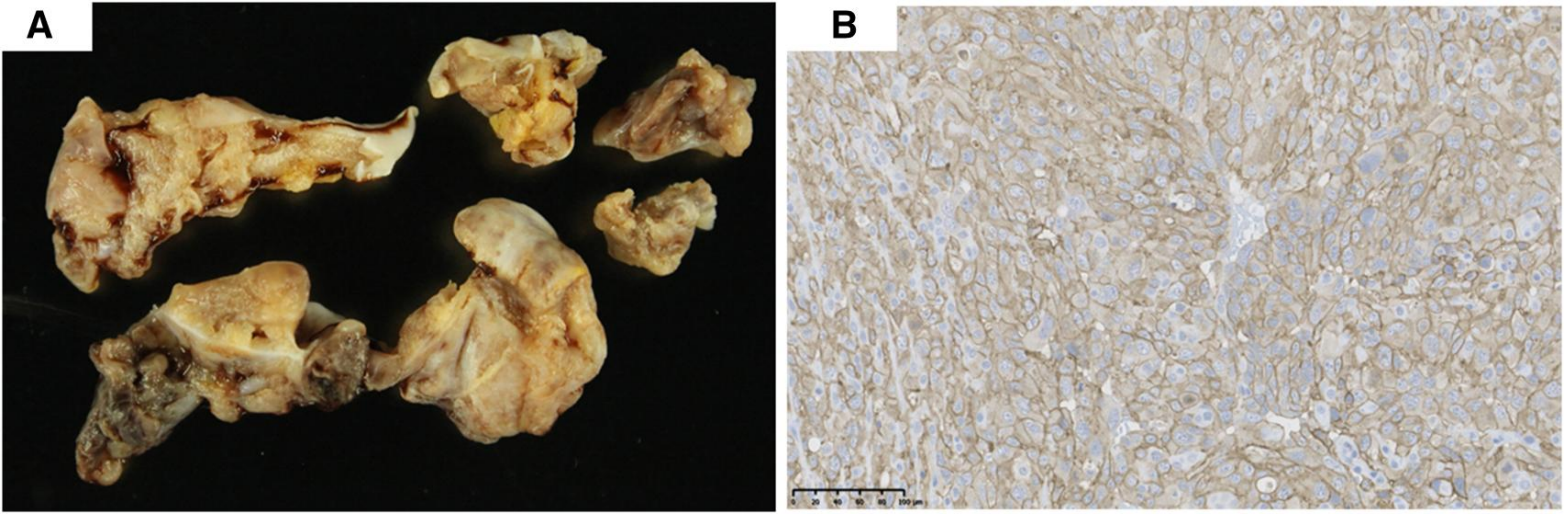
Postoperative pathology. (A) The right atrial tissue at the anastomotic line of the trimmed bovine pericardial patch (macroscopic findings). (B) Immunohistochemically, the atypical cells are diffusely positive for CD31. The lesion comprises diffusely proliferating atypical endothelial cells forming vascular channel-like slits (high-power view).

A median sternotomy revealed a fragile RA tissue with rupture, requiring an immediate complete cardiopulmonary bypass (CPB). The friable tissue was excised, and the 40 × 40 mm RA defect was closed with a bovine pericardial patch (**Video 1**). Anticipating inadequate hemostasis with simple ligation, coronary artery bypass grafting (CABG) was performed from the ascending aorta to the RCA using the great saphenous vein. During weaning from the CPB, transient ventricular fibrillation requiring defibrillation; an IABP was placed and later removed uneventfully on postoperative day 1. On postoperative day 7, the evaluation revealed that the pulmonary nodules had progressed despite the patent graft. Pathological results from the RA tissue led to a definitive PCA diagnosis on postoperative day 9 (**Figure 2**). The patient was discharged on postoperative day 13 and transferred for specialized therapy. Despite the treatment, he died 5 months post-diagnosis due to lung metastatic progression.

## DISCUSSION

A quarter of cardiac tumours are malignant, with approximately 40% of them being PCAs. PCA prognosis is poor, with a median survival of 5.2 months^1^ and usually non-specific clinical findings. Primary cardiac angiosarcomas tend to occur in relatively young adults,^2^ and their initial PCA manifestations comprise cardiac tamponade or heart inflow obstruction.^3^ Primary cardiac angiosarcoma commonly arises in the RA^3^ and could be fatal in cases with RA perforation, as observed in the case of our patient. Negative preoperative pericardial fluid cytology results cannot always definitively rule out cardiac malignancy.^4^ The tumour could not be identified using computed tomography since it merged with the RA and ruptured, with no traces observed during intraoperative exploration. Although coronary artery bypass grafting was performed on the RCA, cardiac function was affected both by the feeder artery ligation and hypotension due to bleeding from the RA rupture before CPB initiation. An intra-aortic balloon pump was introduced due to the refractory ventricular fibrillation that occurred when the patient was weaned off CPB.

Surgical resection, followed by chemotherapy, is the definitive diagnosis and treatment for PCA and is expected to improve the long-term prognosis. Early surgery should be considered based on the results of appropriate imaging modalities, such as cardiac magnetic resonance imaging^5^ and positron emission tomography. According to Look Hong et al, median overall survival for PCA was 13 months, with poorer outcomes in metastatic cases (6 vs 19.5 months).^2^ Complete resection prolonged survival (17 vs 5 months; *P* = .01), and adjuvant therapy might offer modest benefits.

## CONCLUSION

In similarly unique cases, the preoperative suspicion of neoplastic lesions might not be feasible. Unfortunately, their destructive characteristics of such lesions could induce rupture. Therefore, malignancy should be considered in cases of cardiac tamponade of unknown cause in relatively young patients, and an active investigation is warranted.

## Data Availability

Data supporting the findings of this study are available within the article.
